# Models for energy utilization of feeds for nursery and growing pigs based on conventional and multi-sample simultaneous *in vitro* procedures

**DOI:** 10.5713/ab.260384

**Published:** 2026-07-27

**Authors:** Yeojin An, Noa Park, Beob Gyun Kim

**Affiliations:** 1 Department of Animal Science, Konkuk University, Seoul, Korea; 2 Monogastric Animal Feed Research Institute, Konkuk University, Seoul, Korea

**Keywords:** Energy Utilization, *In Vitro*, Nursery Pigs, Prediction Equations, Swine

## Abstract

**Objective:**

The objective of the present study was to develop prediction equations for energy digestibility and metabolizability in feedstuffs and diets for nursery and growing pigs based on *in vitro* total tract disappearance using the conventional procedure determined with the flask method (IVTTD_flask_) and the multi-sample simultaneous method (IVTTD_Daisy_) and chemical composition.

**Methods:**

Fourteen feedstuffs and 18 diets for nursery pigs and 35 feedstuffs and 42 diets for growing pigs were analyzed for gross energy, ether extract, crude protein (CP), ash, amylase-treated neutral detergent fiber (aNDF), and acid detergent fiber. *In vitro* procedures were used to determine IVTTD_flask_ and IVTTD_Daisy_ of dry matter (DM) by simulating digestion and absorption in the stomach, small intestine, and large intestine of pigs. Energy utilization of the feeds in nursery and growing pigs was from in-house data.

**Results:**

The most suitable equations for energy utilization (%) were: energy digestibility_nursery_ = 0.41×IVTTD_Daisy_ of DM–0.28×aNDF+54.0 (root mean square error [RMSE] = 3.12, R^2^ = 0.91, and p<0.001); energy metabolizability_nursery_ = 0.38×IVTTD_Daisy_ of DM–0.25×aNDF–0.09×CP+54.6 (RMSE = 1.39, R^2^ = 0.98, and p<0.001); energy digestibility_growing_ = 0.26×IVTTD_Daisy_ of DM–0.36×aNDF+69.2 (RMSE = 4.54, R^2^ = 0.83, and p<0.001); energy metabolizability_growing_ = 0.26×IVTTD_Daisy_ of DM–0.33×aNDF–0.09×CP+68.2 (RMSE = 4.77, R^2^ = 0.80, and p<0.001), where all nutrients were in % on a DM basis.

**Conclusion:**

The energy utilization by nursery and growing pigs can be estimated using *in vitro* total tract disappearance of DM and chemical composition in feedstuffs and diets.

## INTRODUCTION

Energy is one of the most fundamental components for maintaining body functions and facilitating growth of pigs [1–3]. Swine feed tables provide hierarchical energy values including gross energy (GE), digestible energy, metabolizable energy, and net energy [4]. Energy values in feedstuffs and diets can be determined through animal experiments [5,6], but *in vivo* experiments are laborious and expensive [7–9]. As a more time-efficient and cost-effective alternative to *in vivo* experiments, the conventional *in vitro* procedure has been adopted in which each feed sample is processed and incubated in an individual flask to determine the nutritive values of pig feeds [7,8]. To improve analysis efficiency, the Daisy^II^ incubator (D200I; ANKOM Technology) procedure has been recently developed, which allows simultaneous incubation of multiple samples in a jar [10,11]. Energy utilization of swine feeds can be estimated using prediction equations based on the *in vitro* total tract disappearance (IVTTD) of dry matter (DM) as an independent variable [7,10,12].

Nursery pigs have relatively underdeveloped digestive and absorptive capacities compared with growing pigs [13,14]. The physiological differences between nursery and growing pigs can affect energy utilization [14,15]. Therefore, the influence of growth stage of pigs on the energy utilization of feedstuffs and diets needs to be considered when formulating swine diets [16]. However, previously developed prediction equations were based on the IVTTD of DM for growing pigs without consideration of growth stages [7,10,12]. Therefore, the objective of the present study was to develop prediction equations for estimating energy digestibility and metabolizability in feeds for nursery and growing pigs based on chemical composition in addition to IVTTD determined with the flask method (IVTTD_flask_) or the multi-sample simultaneous method (IVTTD_Daisy_).

## MATERIALS AND METHODS

### Test feeds and *in vivo* data

Fourteen feedstuffs and 18 diets for nursery pigs used in 4 experiments [5,6,14,17] and 35 feedstuffs and 42 diets for growing pigs used in 5 experiments [8,10,11,14,18] were obtained from Konkuk University (Seoul, Republic of Korea; Tables 1–3). The data used in the present study were obtained from experiments conducted using Landrace×Yorkshire or Landrace×Yorkshire×Duroc crossbred barrows. The initial body weight ranged from 7.0±0.8 kg to 12.7±1.3 kg for nursery pigs and from 26.2±2.6 kg to 57.5±5.7 kg for growing pigs. The data consisted of energy utilization and chemical composition of feedstuffs and diets used in animal experiments. The metabolizability of 6 feedstuffs and 5 diets in Oh et al. [18] was calculated using the prediction equation developed by Sung and Kim [19].

### Conventional *in vitro* procedure

The IVTTD_flask_ of DM in 49 test feedstuffs and 60 diets was determined based on Boisen and Fernández [7]. The procedure consisted of 3 steps, and each step simulated digestion in the stomach, the small intestine, and the large intestine of pigs, respectively. In the first step, 0.5 g of the sample was placed in a 100-mL conical flask using 1 mL of pepsin solution (25 mg/mL; ≥250 units/mg solid, P7000, Pepsin from porcine gastric mucosa; Sigma-Aldrich) under pH 2.0 for 2 h at 39°C. In the second step, the sample in the flask was added with 1 mL of pancreatin solution (100 mg/mL; 4×USP, P1750, Pancreatin from porcine pancreas; Sigma-Aldrich) for incubation at pH 6.8, for 4 h at 39°C. Pancreatin solution was centrifuged at 4,000×g for 2 min and only supernatant was used. In the third step, the sample in the flask was added with 10 mL of 0.2 *M* EDTA solution. After adjusting the pH of the mixture to 4.8, a substitution of microbial enzyme, 0.5 mL of Viscozyme (V2010; Sigma-Aldrich), was added for incubation for 18 h at 39°C. After incubation, the samples were then filtered, and the undigested residues were collected and dried for 6 h at 130°C. During the 3-step *in vitro* procedure, a blank was included to correct the DM contents in the residues that did not originate from the diets or feedstuffs.

### Multi-sample simultaneously *in vitro* assay

The IVTTD_Daisy_ of DM in 49 test feedstuffs and 60 diets was determined using procedures reported in previous studies described by Lee et al. [11]. The first step of the procedure, briefly, 0.5 g of the sample was placed in a pre-weighed F57 filter bag (ANKOM Technology) and digested using pepsin solution containing 25 mg of pepsin (powder from porcine gastric mucosa, ≥250 units/mg solid; P7000; Sigma-Aldrich) at pH 6.0 for 2 h at 39°C. During the second step, pancreatin solution containing 100 mg of pancreatin (from porcine pancreas, 4×USP specifications; P1750, Sigma-Aldrich) was added to the sample in the jar for incubation at pH 6.8 for 14 h at 39°C. The pancreatin solution was centrifuged at 4,000×g for 2 min and only the supernatant was used. In the third step, 10 mL of a 0.2 *M* EDTA solution was added to the sample in the jar. After adjusting the pH of the mixture to 4.8, 0.5 mL of Viscozyme (V2010; Sigma-Aldrich) was added to each sample for incubation for 18 h at 39°C. At the end of the procedure, all bags were rinsed twice with distilled water. Then, the bags were rinsed with 96% ethanol and 99.5% acetone twice in the jars to soak and rinse the sample bags. Lastly, the bags were pat-dried with a paper towel (Wypall L25; Yuhan-Kimberly), placed in a forced-air drying oven to be dried until constant weight, and weighed. During the *in vitro* procedure, a blank bag was included to correct the DM contents in the residues that did not originate from the diets or test feedstuffs.

### Chemical analysis

Ether extract (method 920.39), crude protein (CP; method 990.03), and ash (method 942.05) in feedstuffs and diets were determined as described in AOAC International [20]. Gross energy concentrations in the feedstuffs and diets were determined using bomb calorimetry (Parr 6400; Parr Instruments). Additionally, amylase-treated neutral detergent fiber (aNDF; method 2002.04) and acid detergent fiber (ADF; method 973.18) contents in feedstuffs and diets were analyzed as described in AOAC International [20].

### Calculation

The IVTTD_flask_ and IVTTD_Daisy_ of DM were calculated using the following equation [10]:


(1) $${\text{IVTTD}}_{\text{flask}}\;\text{or }{\text{IVTTD}}_{\text{Daisy}}\;\text{of DM }(\%)=({\text{DM}}_{\text{sample}}-[{\text{DM}}_{\text{residue}}-{\text{DM}}_{\text{blank}}])÷{\text{DM}}_{\text{sample}}\times 100$$

where DM_sample_ (g) is the amount of DM in the diets or feedstuffs, DM_residue_ (g) is the amount of DM residue after *in vitro* digestion procedures, and DM_blank_ (g) is the amount of DM residue after *in vitro* digestion procedures in the blank.

### Statistical analysis

Before the statistical analysis of the obtained dataset, outliers were removed for the final statistical analysis. Three observations with Cook’s distance values greater than Fox’s criterion (0.129) in the nursery pig dataset and one observation exceeding the criterion (0.052) in the growing pig dataset were excluded [21]. The correlation coefficient among energy utilization, IVTTD, and chemical compositions in diets and feedstuffs were determined by the CORR procedure of SAS (SAS Institute). Prediction equations for energy utilization were developed by the REG procedure of SAS. A coefficient of determination (R^2^) was an indicator to define the best-fit equations. The alpha level used for determining statistical significance was 0.05.

## RESULTS

In the 14 feedstuffs for nursery pigs, the energy digestibility ranged from 54.2% to 95.3%, and the energy metabolizability ranged from 53.5% to 88.2%. The ranges of IVTTD_flask_ of DM and IVTTD_Daisy_ of DM were from 35.3% to 94.8% and 38.5% to 96.8%, respectively (Table 1). The CP concentration of the feedstuffs for nursery pigs ranged from 8.4% to 70.9% DM, and aNDF ranged from 3.3% to 76.2% DM. In the 18 diets for nursery pigs, the energy digestibility ranged from 74.6% to 90.1%, and the energy metabolizability ranged from 72.0% to 88.2%. The ranges of IVTTD_flask_ of DM and IVTTD_Daisy_ of DM were from 66.5% to 90.5% and 68.1% to 91.1%, respectively. The CP concentration of the nursery diets ranged from 8.1% to 26.8% DM, and aNDF ranged from 7.0% to 31.4% DM. In the 35 feedstuffs for growing pigs, the energy digestibility ranged from 24.3% to 100.6%, and the energy metabolizability ranged from 21.9% to 96.6%. The ranges of IVTTD_flask_ of DM and IVTTD_Daisy_ of DM in the feedstuffs for growing pigs ranged from 28.7% to 99.6% and 36.7% to 99.6%, respectively. The CP concentration of the feedstuffs for growing pigs ranged from 4.1% to 85.9% DM, and aNDF ranged from −0.3% to 72.6% DM. In the 42 diets for growing pigs, energy digestibility ranged from 67.0% to 96.3%, and energy metabolizability ranged from 63.9% to 93.6%. The ranges of IVTTD_flask_ of DM and IVTTD_Daisy_ of DM in the growing diets were from 71.3% to 98.6% and 74.1% to 98.6%, respectively. The concentration of CP in the growing diets ranged from 0.1% to 34.7% DM, and aNDF ranged from 0.2% to 34.2% DM.

In the feedstuffs and diets for nursery pigs, the energy digestibility and metabolizability were positively correlated with IVTTD_flask_ of DM (r = 0.86, p<0.001 and r = 0.80, p<0.001, respectively; Table 4) and IVTTD_Daisy_ of DM (r = 0.92, p<0.001 and r = 0.88, p<0.001, respectively). The energy digestibility and metabolizability of nursery pigs were negatively correlated with aNDF (r = −0.93, p<0.001 and r = −0.93, p<0.001, respectively). In the feedstuffs and diets for growing pigs, the energy digestibility and metabolizability were positively correlated with IVTTD_flask_ of DM (r = 0.86, p<0.001 and r = 0.83, p<0.001, respectively; Table 5) and IVTTD_Daisy_ of DM (r = 0.87, p<0.001 and r = 0.84, p<0.001, respectively). The energy digestibility and metabolizability of growing pigs were negatively correlated with aNDF (r = −0.90, p<0.001 and r = −0.87, p<0.001, respectively).

Based on R^2^, the best-fit models for the energy digestibility (%) and metabolizability (%) for nursery pigs were equations 4 and 10, respectively (Table 6): energy digestibility_nursery_ = 0.41×IVTTD_Daisy_ of DM–0.28×aNDF+54.0 (root mean square error [RMSE] = 3.12, R^2^ = 0.91, and p<0.001); energy metabolizability_nursery_ = 0.38×IVTTD_Daisy_ of DM–0.25×aNDF–0.09× CP+54.6 (RMSE = 1.39, R^2^ = 0.98, and p<0.001), where all nutrients were in % on a DM basis. In the growing pigs, best-fit models for the energy digestibility (%) and metabolizability (%) were equations 14 and 20 (Table 7): energy digestibility_growing_ = 0.26×IVTTD_Daisy_ of DM–0.36×aNDF+69.2 (RMSE = 4.54, R^2^ = 0.83, and p<0.001); energy metabolizability_growing_ = 0.26× IVTTD_Daisy_ of DM–0.33×aNDF–0.09×CP+68.2 (RMSE = 4.77, R^2^ = 0.80, and p<0.001), where all nutrients were in % on a DM basis.

## DISCUSSION

Pig diets consist of various feedstuffs at different inclusion levels to meet goals for nutrient requirements, cost-efficiency, and functionality, resulting in differences in energy utilization among diets [22]. To maximize growth performance of pigs, an accurate determination of the energy utilization of feedstuffs and diets is essential [12]. Energy utilization in swine feeds can be estimated using prediction equations based on IVTTD of DM [12,23]. When developing prediction equations, a wide range of independent variables increases their applicability to feedstuffs and diets [24,25]. However, the ranges of the nutrient concentrations used for developing previous equations were rather narrow [12,23]. To overcome this limitation, the feedstuffs and diets with wider ranges of aNDF were employed in the present work. In addition, prediction equations for energy utilization have been developed separately for nursery pigs and growing pigs considering the different digestive capacities between nursery and growing pigs [14,15]. The use of IVTTD of DM as the sole independent variable in the prediction equation may not fully reflect complex gastrointestinal physiology of pigs where varying nutrient composition of feedstuffs influence *in vivo* digestion [10,26]. Previous studies have reported that fibers in feedstuffs potentially lead to differences between *in vitro* and *in vivo* digestibility values [27,28]. One possible explanation for this discrepancy is that dietary fibers accelerate the passage rate of digesta through the gastrointestinal tract, thereby reducing nutrient digestibility *in vivo* [17,29]. However, *in vitro* procedures apply standardized conditions regardless of the fiber content of feedstuffs and therefore cannot adequately reflect peristalsis and digesta passage rate, which may result in an overestimation of *in vitro* digestibility [7]. This limitation may be at least partially overcome by the use of fiber as independent variable in addition to *in vitro* digestibility in the models for estimating *in vivo* digestibility.

Enzyme activities in the simulated large intestinal fluid during the *in vitro* procedures are critical for successfully mimicking digestion in the large intestine of pigs [30]. In the present study, Viscozyme was used as a substitute for microbial carbohydrase to simulate fiber degradation in the large intestine [7]. However, Viscozyme may not fully reflect the microbial fermentation in the hindgut of pigs and the influence of microbiota on digesta retention time as the diversity of microbiota and enzyme activities would be affected by the substrates that reach the hindgut [31]. Therefore, the addition of nutrient concentrations as independent variables for estimating *in vivo* digestibility may partially account for discrepancies between *in vitro* and *in vivo* digestibility values and thereby improve the prediction accuracy of energy utilization in pigs.

*In vivo* energy utilization was positively correlated with IVTTD_flask_ and IVTTD_Daisy_ of DM, which is consistent with previous observations [10,12]. This is likely due to the fact that large portion of energy digestibility is dependent on DM digestibility [5,17]. The negative correlations between aNDF and energy utilization are consistent with previous studies [19,32]. Pigs have limited ability to digest fiber within their gastrointestinal tract because of the insufficient presence of enzymes required for fiber degradation [33,34]. Additionally, dietary fiber may increase the passage rate of digesta and interfere with digestive enzyme activity in the gastrointestinal tract, thereby reducing the utilization of energy and nutrients [33,34]. The aNDF was found to be the most representative among crude fiber, aNDF, ADF, soluble dietary fiber, and insoluble dietary fiber in a previous study due to fewer analysis errors in the detergent fiber procedure [25].

Although the CP content was not correlated with energy metabolizability, including CP as an independent variable together with aNDF in the prediction equations improved the R^2^. This may be explained by the fact that variation in energy metabolizability was largely driven by aNDF due to its strong influence on energy digestibility, thereby reducing the relative statistical contribution of CP. Because metabolizable energy is defined as digestible energy minus urinary and gaseous energy losses [4], dietary CP can affect energy metabolizability through contribution to urinary energy loss [35–37]. However, the *in vitro* procedure mimics digestion and absorption but does not represent post-absorptive nutrient and energy utilization [10]. Therefore, including CP as an independent variable can account for post-absorptive nitrogen metabolism and urinary energy losses not captured by *in vitro* procedure [38], thereby improving the accuracy of energy metabolizability prediction models.

Physiological differences between developmental stages in pigs can lead to variations in energy utilization [13,16]. Nursery pigs have lower energy digestibility than growing pigs due to their less developed digestive systems and limited secretion of digestive enzymes [13]. Additionally, because growing pigs have a greater fermentation capacity due to the more developed large intestine, growing pigs can produce more energy-providing substances such as volatile fatty acids, resulting in higher energy availability from fibers [16,39]. Nursery pigs have less digestive capability for energy compared with growing pigs particularly when a diet contains high concentrations of fiber [15]. Thus, the influence of dietary fiber concentrations on the variability of energy digestibility would be greater in nursery pigs than in growing pigs. This may explain the stronger negative correlation between energy utilization and fiber components in nursery pigs observed in the present experiment. Therefore, the consideration of specific growth stages of pigs would be necessary when developing prediction equations for estimating energy utilization.

The reason for the greater R^2^ observed when using IVTTD_Daisy_ of DM as an independent variable compared with IVTTD_flask_ of DM remains unclear. This difference may be due to variations in the types of feedstuffs used during the development of each *in vitro* procedure. The multi-sample simultaneous *in vitro* method was developed primarily using plant-origin feedstuffs [11] whereas the conventional *in vitro* procedure included some animal-origin feedstuffs such as fish meal, meat and bone meal, and animal fat [7]. Because the present work mainly included plant-origin feedstuffs and did not use animal-based feedstuffs, the multi-sample simultaneous *in vitro* procedure may have been more compatible with the experimental diets employed in the present experiment.

## CONCLUSION

The prediction equations developed in the present work can be used for predicting energy utilization in feedstuffs and diets based on IVTTD in nursery and growing pigs. The most suitable equations for energy utilization (%) were: energy digestibility_nursery_ = 0.41×IVTTD_Daisy_ of DM–0.28×aNDF+ 54.0; energy metabolizability_nursery_ = 0.38×IVTTD_Daisy_ of DM–0.25×aNDF–0.09×CP+54.6; energy digestibility_growing_ = 0.26×IVTTD_Daisy_ of DM–0.36×aNDF+69.2; energy metabolizability_growing_ = 0.26×IVTTD_Daisy_ of DM–0.33×aNDF–0.09× CP+68.2, where all nutrients were in % on a DM basis. Further studies are warranted to validate the present equations and to develop prediction equations for net energy in the future. In addition, the development of separate prediction equations for different dietary protein groups should also be considered.

## Figures and Tables

**Table 1:** The variability of energy utilization and chemical components in feedstuffs and diets in the database, dry matter (DM) basis

	Feedstuffs					Diets				
	
	n	Mean	Min.	Max.	SD	n	Mean	Min.	Max.	SD
Nursery pigs										
Energy digestibility (%)	14	80.6	54.2	95.3	14.7	18	84.7	74.6	90.1	4.4
Energy metabolizability (%)	14	77.1	53.5	88.2	11.7	18	82.2	72.0	88.2	4.5
IVTTD_flask_ of DM (%)	14	79.8	35.3	94.8	18.4	18	82.2	66.5	90.5	6.6
IVTTD_Daisy_ of DM (%)	14	79.7	38.5	96.8	19.9	18	83.4	68.1	91.1	6.7
Gross energy (kcal/kg)	14	4,899	4,339	6,830	653	18	4,381	4,206	5,115	208
Ether extract (%)	14	6.1	0.4	47.6	12.2	18	3.9	3.0	5.6	0.7
Crude protein (%)	14	39.7	8.4	70.9	21.9	18	18.9	8.1	26.8	5.4
Ash (%)	14	6.3	1.5	12.9	2.8	18	5.7	4.2	6.7	0.8
aNDF (%)	14	23.6	3.3	76.2	23.8	18	14.5	7.0	31.4	6.5
Acid detergent fiber (%)	14	12.9	1.8	50.7	15.2	18	6.5	2.0	18.9	4.4
Growing pigs										
Energy digestibility (%)	35	74.4	24.3	100.6	5.8	42	84.5	67.0	96.3	5.8
Energy metabolizability (%)	35	71.6	21.9	96.6	5.8	42	82.1	63.9	93.6	5.8
IVTTD_flask_ of DM (%)	35	73.6	28.7	99.6	7.6	42	83.3	71.3	98.6	7.6
IVTTD_Daisy_ of DM (%)	35	72.7	36.7	99.6	6.9	42	85.3	74.1	98.6	6.9
Gross energy (kcal/kg)	35	4,649	4,149	5,429	348	42	4,395	4,013	4,830	185
Ether extract (%)	35	4.9	−0.2	22.5	1.9	42	5.0	1.0	11.4	1.9
Crude protein (%)	35	24.8	4.1	85.9	5.5	42	17.1	0.1	34.7	5.5
Ash (%)	35	5.9	0.9	12.9	1.0	42	5.6	3.7	7.8	1.0
aNDF (%)	35	32.6	−0.3	72.6	8.5	42	17.4	0.2	34.2	8.5
Acid detergent fiber (%)	35	17.6	0.2	51.4	5.1	42	7.4	0.7	21.9	5.1

The values were obtained from 4 experiments with nursery pigs [5,6,14,17] and from 5 experiments with growing pigs [8,10,11,14,18].

SD, standard deviation; IVTTD_flask_, *in vitro* total tract disappearance determined using the flask; IVTTD_Daisy_, *in vitro* total tract disappearance determined using the Daisy^II^ incubator; aNDF, amylase-treated neutral detergent fiber.

**Table 2:** *In vitro* total tract disappearance (IVTTD, %) of dry matter (DM) and energy utilization (%) of feedstuffs with nursery pigs

Feedstuffs	n	IVTTD_flask_ of DM	IVTTD_Daisy_ of DM	Energy digestibility	Energy metabolizability
Cashew nut	1	94.4	96.8	90.7	88.1
Copra meal	1	70.5	66.2	70.1	66.7
Corn	1	85.3	87.7	88.0	86.3
Corn derived protein	1	81.0	80.5	88.3	76.9
Fermented soybean meal	3	92.1	93.9	94.5	85.7
Palm kernel expellers	1	39.2	38.0	57.2	53.5
Rapeseed meal	1	75.9	73.7	75.1	70.3
Soybean hulls	1	53.4	47.1	54.2	53.7
Soybean meal	2	94.2	94.9	84.8	79.3
Wheat bran	1	62.7	57.7	62.4	60.1
Soy protein concentrate	1	94.6	95.7	90.9	83.9

The values were obtained from 4 experiments with nursery pigs [5,6,14,17].

IVTTD_flask_, IVTTD determined using the flask; IVTTD_Daisy_, IVTTD determined using the Daisy^II^ incubator.

**Table 3:** *In vitro* total tract disappearance (IVTTD, %) of dry matter (DM) and energy utilization (%) of feedstuffs with growing pigs

Feedstuffs	n	IVTTD_flask_ of DM	IVTTD_Daisy_ of DM	Energy digestibility	Energy metabolizability
Barley	1	87.4	84.9	80.0	77.2
Copra meal	1	72.2	71.7	67.7	65.4
Corn	2	84.7	87.9	88.2	86.8
Corn DDGS	1	62.3	66.0	71.1	67.2
Corn gluten feed	4	62.8	65.9	61.3	57.9
Lupin kernel	1	96.0	93.0	87.4	84.6
Lupin hulls	1	60.0	52.3	55.9	54.7
Palm kernel expellers	2	44.2	41.2	65.3	63.5
Pea protein concentrate	1	92.1	95.2	96.4	88.7
Rapeseed meal	2	76.8	75.8	68.7	64.1
Rice	1	97.0	99.2	99.5	96.6
Rice bran	3	73.0	62.6	72.9	71.3
Skimmed milk powder	1	99.6	99.6	97.1	92.2
Soy protein isolate	1	95.4	98.6	100.6	91.7
Soybean hulls	3	52.4	55.1	63.3	64.0
Soybean meal	3	93.9	93.1	87.1	82.9
Sugar beet pulp	1	79.7	80.2	70.7	69.9
Wheat	2	90.8	89.6	89.2	86.3
Wheat bran	4	66.0	58.7	67.5	65.8

The values were obtained from 5 experiments with growing pigs [8,10,11,14,18].

IVTTD_flask_, IVTTD determined using the flask; IVTTD_Daisy_, IVTTD determined using the Daisy^II^ incubator; DDGS, distillers dried grains with solubles.

**Table 4:** Correlation coefficients among energy utilization, *in vitro* total tract disappearance (IVTTD) of dry matter (DM), and chemical components in feedstuffs and diets in nursery pigs (n = 32)

Item	Energy digestibility	Energy metabolizability	IVTTD_flask_ of DM	IVTTD_Daisy_ of DM	EE	CP	Ash	aNDF
Energy metabolizability	0.89^***^							
IVTTD_flask_ of DM	0.86^***^	0.80^***^						
IVTTD_Daisy_ of DM	0.92^***^	0.88^***^	0.95^***^					
EE	0.10	0.18	0.17	0.18				
CP	0.38^*^	−0.04	0.37	0.32	−0.14			
Ash	−0.16	−0.30	0.04	−0.06	−0.43^*^	0.39^*^		
aNDF	−0.93^***^	−0.93^***^	−0.83^***^	−0.87^***^	−0.04	−0.17	0.14	
ADF	−0.87^***^	−0.91^***^	−0.75^***^	−0.82^***^	−0.04	−0.03	0.25	0.95^***^

* p<0.05 and

*** p<0.001.

IVTTD_flask_, IVTTD determined using the flask; IVTTD_Daisy_, IVTTD determined using the Daisy^II^ incubator; EE, ether extract; CP, crude protein; aNDF, amylase-treated neutral detergent fiber; ADF, acid detergent fiber.

**Table 5:** Correlation coefficients among energy utilization, *in vitro* total tract disappearance (IVTTD) of dry matter (DM), and chemical components in feedstuffs and diets in growing pigs (n = 77)

Item	Energy digestibility	Energy metabolizability	IVTTD_flask_ of DM	IVTTD_Daisy_ of DM	EE	CP	Ash	aNDF
Energy metabolizability	0.99^***^							
IVTTD_flask_ of DM	0.86^***^	0.83^***^						
IVTTD_Daisy_ of DM	0.87^***^	0.84^***^	0.93^***^					
EE	−0.18	−0.16	−0.23^*^	−0.33^**^				
CP	0.16	0.06	0.22	0.21	−0.16			
Ash	−0.32^**^	−0.37^**^	−0.19	−0.23^*^	0.33^**^	0.32^**^		
aNDF	−0.90^***^	−0.87^***^	−0.90^***^	−0.88^***^	0.11	−0.19	0.17	
ADF	−0.75^***^	−0.71^***^	−0.79^***^	−0.77^***^	0.04	−0.15	0.08	0.92^***^

* p<0.05,

** p<0.01, and

*** p<0.001.

IVTTD_flask_, IVTTD determined using the flask; IVTTD_Daisy_, IVTTD determined using the Daisy^II^ incubator; EE, ether extract; CP, crude protein; aNDF, amylase-treated neutral detergent fiber; ADF, acid detergent fiber.

**Table 6:** Prediction equations for energy utilization (%) in feedstuffs and diets based on *in vitro* total tract disappearance (IVTTD, %) of dry matter (DM) and chemical compositions (% DM) in nursery pigs (n = 32)

No.	Equation	RMSE	R_2_	p-value
1	Digestibility_nursery_ = 0.71×IVTTD_flask_ of DM+24.7	4.46	0.82	<0.001
2	Digestibility_nursery_ = 0.69×IVTTD_Daisy_ of DM+25.8	3.35	0.90	<0.001
3	Digestibility_nursery_ = 0.13×IVTTD_flask_ of DM–0.53×aNDF+81.1	3.58	0.89	<0.001
4	Digestibility_nursery_ = 0.41×IVTTD_Daisy_ of DM–0.28×aNDF+54.0	3.12	0.91	<0.001
5	Metabolizability_nursery_ = 0.61×IVTTD_flask_ of DM+29.6	3.37	0.85	<0.001
6	Metabolizability_nursery_ = 0.59×IVTTD_Daisy_ of DM+31.0	2.52	0.92	<0.001
7	Metabolizability_nursery_ = 0.08×IVTTD_flask_ of DM–0.49×aNDF+81.2	2.30	0.93	<0.001
8	Metabolizability_nursery_ = 0.26×IVTTD_Daisy_ of DM–0.33×aNDF+64.0	2.00	0.95	<0.001
9	Metabolizability_nursery_ = 0.20×IVTTD_flask_ of DM–0.41×aNDF–0.08×CP+71.9	1.99	0.95	<0.001
10	Metabolizability_nursery_ = 0.38×IVTTD_Daisy_ of DM–0.25×aNDF–0.09×CP+54.6	1.39	0.98	<0.001

RMSE, root mean square error; IVTTD_flask_, IVTTD determined using the flask; IVTTD_Daisy_, IVTTD determined using the Daisy^II^ incubator; aNDF, amylase-treated neutral detergent fiber; CP, crude protein.

**Table 7:** Prediction equations for energy utilization (%) in feedstuffs and diets based on *in vitro* total tract disappearance (IVTTD, %) of dry matter (DM) and chemical compositions (% DM) in growing pigs (n = 77)

No.	Equation	RMSE	R^2^	p-value
11	Digestibility_growing_ = 0.73×IVTTD_flask_ of DM+23.0	5.54	0.75	<0.001
12	Digestibility_growing_ = 0.66×IVTTD_Daisy_ of DM+27.8	5.48	0.75	<0.001
13	Digestibility_growing_ = 0.24×IVTTD_flask_ of DM–0.39×aNDF+70.8	4.65	0.82	<0.001
14	Digestibility_growing_ = 0.26×IVTTD_Daisy_ of DM–0.36×aNDF+69.2	4.54	0.83	<0.001
15	Metabolizability_growing_ = 0.67×IVTTD_flask_ of DM+25.4	5.89	0.68	<0.001
16	Metabolizability_growing_ = 0.61×IVTTD_Daisy_ of DM+29.3	5.72	0.70	<0.001
17	Metabolizability_growing_ = 0.18×IVTTD_flask_ of DM–0.38×aNDF+72.9	5.08	0.77	<0.001
18	Metabolizability_growing_ = 0.23×IVTTD_Daisy_ of DM–0.34×aNDF+68.0	4.95	0.78	<0.001
19	Metabolizability_growing_ = 0.20×IVTTD_flask_ of DM–0.38×aNDF–0.09×CP+73.8	4.94	0.78	<0.001
20	Metabolizability_growing_ = 0.26×IVTTD_Daisy_ of DM–0.33×aNDF–0.09×CP+68.2	4.77	0.80	<0.001

RMSE, root mean square error; IVTTD_flask_, IVTTD of DM determined using the flask; IVTTD_Daisy_, IVTTD of DM determined using the Daisy^II^ incubator; aNDF, amylase-treated neutral detergent fiber; CP, crude protein.

## Data Availability

Upon reasonable request, the datasets of this study can be available from the corresponding author.
